# The role of exosomes in the pathogenesis of Alzheimer’ disease

**DOI:** 10.1186/s40035-017-0072-x

**Published:** 2017-02-02

**Authors:** Tingting Xiao, Weiwei Zhang, Bin Jiao, Chu-Zheng Pan, Xixi Liu, Lu Shen

**Affiliations:** 1 Department of Neurology, Xiangya Hospital, Central South University, Changsha, China; 2 State Key Laboratory of Medical Genetics, Changsha, China; 3 Key Laboratory of Hunan Province in Neurodegenerative Disorders, Central South University, Changsha, China

## Abstract

Exosomes are small vesicles secreted by most cell types including neurons that function in intercellular communication through transfer of their cargo or encapsulate and eliminate unnecessary cellular components and therefore have a broad impact on nerve development, activation and regeneration. In addition, exosomes have been observed to be involved in spreading pathological misfolded proteins, thereby leading to the onset and propagation of disease. Alzheimer disease (AD) is the most common form of dementia and characterized by two types of lesions: amyloid plaques and neurofibrillary tangles. Accumulating evidence has demonstrated that exosomes are associated with amyloid precursor (APP) and Tau proteins and play a controversial role in Alzheimer’s disease process. In this review, we will discuss the role of exosomes in the metabolism and secretion of APP and Tau proteins and their subsequent impact on AD pathogenesis.

## Background

According to the 2016 World Alzheimer Report, there are 47 million people living with dementia worldwide [1]. It is estimated that the total worldwide cost of dementia is $818 billion (USD) and is expected to reach $1 trillion (USD) by 2018, thus placing a huge burden on individuals, families, and societies [1]. As the leading cause of dementia, Alzheimer’s disease (AD) accounts for an estimated 60 to 80% of all cases [2]. It is clinically characterized by cognitive impairment, a variety of neuropsychiatric symptoms and the restriction of daily life activities [3]. AD is pathologically defined by the deposits of the protein fragment beta-amyloid (Aβ plaques) outside neurons and twisted fibers of the protein tau that build up inside neurons (NFTs). The cause for most AD cases is still uncovered except for 1 to 5% of cases which develop as a result of mutations in the presenilin1 (*PSEN1*), presenilin2 (*PSEN2*), or amyloid precursor protein (*APP*) genes [4].

Recently, the role of “Prion-like mechanisms” in the pathogenesis of neurodegenerative diseases has attracted more and more attention. It has been suggested that pathologically misfolded proteins can transfer their conformation to properly folded proteins, thus resulting in the propagation of disease [5]. For instance, plaques and tangles tend to spread through the cortex in a predictable pattern as Alzheimer’s disease progresses [6]. While, the mechanisms underlying the spread of misfolded proteins still poorly understood. There are several pathways for signal delivery and material communication between cells, such as synaptic transmission, direct communication trough gap junction and paracrine signaling [7]. Among these hypotheses, accumulating evidence supports the idea that exosomes may play as a messenger to participate in cell communication and contribute to this lesions spreading [8, 9].

Exosomes were first reported in reticulocytes and considered to function in the disposal of unnecessary cellular components [10, 11]. Exosomes are nanosized extracellular vesicles (generally 50-100 nm diameter) that can be released by nearly all cell types, including neuronal cells [12]. The exosomes’ molecular contents include proteins, lipids and genetic material. Exosomes are released in bodily fluids and shuttle molecules for long distances for the purpose of intercellular communication. Exosomes have been reported to implicate in the spread of pathological proteins involved in neurodegenerative diseases, such as AD, Parkinson’s disease (PD) and the prion diseases. APP, β- secretase, γ- secretase has been detected in exosomes, what’s more, exosomal proteins such as Alix and Flotillin were also found to be accumulated in the plaques of AD patient brains [13].

In this review, we will discuss role of exosomes in the metabolism and secretion of APP and Tau proteins and the subsequent impact on AD pathogenesis.

## Biogenesis of exosomes

Exosomes are small membrane vesicles that are generated via endocytic pathways [14, 15]. Inward budding of the plasma membrane forms small vesicles, which undergo fused together to form the early endosome. Intraluminal vesicles (ILVs) begin to compose through invagination of the limiting endosomal membrane during the maturation process of early endosome. Upon creation, cytoplasmic molecules such as proteins, lipids, and RNAs are encapsuled into the lumen and accumulated within the late endosome, thus forming multi-vesicular bodies (MVBs). There are two fates for MVBs, some of which transport to lysosomes for degradation (dMVBs), while others fuse with the plasma membrane and release ILVs into the extracellular space as exosomes (sMVBs). Compared with the dMVBs which are enriched in bismonoacylglycerophoshate (BMP, LBPA), the sMVBs contain more of ceramides [16, 17]. ILV formation is the key step in exosome biogenesis [18]. The formation of ILVs is mainly regulated by the complex of multi-molecular machinery named Endosomal Sorting Complex Required for Transport (ESCRT) [19, 20]. However, studies have shown that depletion of ESCRT subunits does not totally impair the composition of MVBs, which indicate that other mechanisms may exist in the process of ILVs formation [21]. It suggested that proper level of lipids and tetraspanin-enriched micro-domains is needed for MVBs formation [22–25]. Exosome secretion is also regulated by membrane depolarization.

## Molecular contents of exosomes

The molecules within exosomes can be divided into two types: constitutive molecules and cargo molecules. Constitutive molecules are unique to exosomes regardless of the cell type from which they are derived and play an essential role in keeping fundamental structures and functions of exosomes. Cargo molecules, on the other hand, are proteins, lipids and genetic material which are sorted, encapsulated and transported by exosomes. The cargo molecules are variable according to cell origin and the physiological or pathological conditions when exosomes generate. In addition, sorting of molecules into exosomes is thought to be a selective process, since some accumulated factors observed in exosomes are barely detectable in parental cells.

The protein composition of exosomes has been analyzed extensively. Since exosomes are released through the endosome pathway, proteins such as tetraspanins (CD9, CD63, CD81 and CD82), Rab GTPases, flotillin, Alix, TSG101and heat shock proteins (Hsc70, Hsp90) have been all identified in exosomes [26–29]. In addition to constitutive molecules, exosomes with different cell origin carry specific proteins. For example, major histocompatibility complex class II (MHCII) is mainly present on exosomes derived from antigen presenting cells [30]. Cells can also release prions, beta-amyloid peptides, tau protein, misfolded superoxide dismutase-1(SOD1) and alpha-synuclein through exosomes in different pathological and physiological conditions [13, 31–34]. Lipids in exosomes mainly work as regulating exosomal sorting of small RNAs and proteins [35, 36].

In addition to proteins and lipids, genetic materials are also found in exosomes, such as DNA, mRNA, miRNA, ribosomal RNA (rRNA), circular RNA, and long noncoding RNA (lnRNA) [37–42]. Among them, small RNA (<30 nucleotides) account for a large proportion, making up >50% of all exosomal RNA species [38, 40, 43]. However, a few studies have shown different results in which ribosomal RNA, in particular 28S and 18S rRNA subunits, were found to be the major class of RNA in exosomes [39]. These conflicting results may be due to the purity of the exosome preparation and differences in cell origin. It has been shown that exosome RNA is functional. Valadi and colleagues detected the expression of mouse proteins after transfer of mouse exosomal RNA to human mast cells [43]. What’ more previous studies showed that miR-222 transferred through exosome was able to increase tumor malignancy in melanoma through suppression of p27Kip1 expression and induction of the PI3K/AKT pathway [44].

## Function of exosomes in the central nervous system (CNS)

Exosomes can be released by most cell types in the CNS, such as neurons, astrocytes, oligodendrocytes and microglia, and participate in regulating neuronal development, regeneration, and modulation of synaptic functions [45–47]. The main physiological roles of exosomes include eliminating cellular waste, regulating immune response and communicating between neural cells [20, 48, 49]. Once released into extracellular space, exosomes act as messengers, can be captured by neighboring cells or internalized by cells with a certain distance, or enter body fluids and taken up by different tissues [50]. There are several ways for signal transduction mediated by exosomes, such as receptor-ligand pathway, endocytosis and phagocytosis [51]. Because of the double membrane structure, exosomes pathway may have a higher efficiency in transfer substance.

In CNS, both glia and neuron secrete exosomes is regulated by glutamate in a certain degree. It has been hypothesized that exosomes can be served as messenger to mediate the communication between neuron and glia. While, as the reported, exosomes derived from neurons can only be captured by neurons, but not glia. It is interesting to note exosomes secreted by neuroblastoma cells can bind with both of neurons and glial cells. It demonstrate that cell communication mediated by exosomes has cell- selectivity [52].

In addition, the function of exosomes may be variable among different cell origins. Evidence shows that exosomes derived from N2a cells or isolated from human cerebrospinal fluid can abolish the synaptic plasticity disruption caused by both synthetic and AD brain-derived Aβ [53]. However, Asai and colleagues observed that exosomes derived from microglia can spread tau protein, and inhibiting exosome synthesis significantly reduced tau propagation in vitro and in vivo [47].

Except the physiological function, the role of exosomes in spreading toxic proteins and inducing the propagation of diseases such as AD has been discussed extensively.

## Impact of exosomes on amyloidogenic processing of APP

The major component of amyloid deposits is small peptides, 39–43 amino acids in length named Aβ, which is derived from a sequence of successive cleavages of APP [54]. APP is a type-I transmembrane glycoproteins. Three secretases termed α, β and γ-secretases are involved in the metabolism of APP. In the amyloidogenic pathway, upon cleavage by β-secretase (BACE-1) and γ-secretase, a large soluble ectodomain fragment (sAPP-β), membrane-bound C-terminal fragment (β-CTF), a small APP intracellular fragment (AICD) and Aβ peptides are produced [55, 56]. In the non-amyloidogenic pathway, APP is initially cleaved at the α-secretase site, generating sAPP-α and α-CTF. The latter is further processed by the γ-secretase complex, releasing AICD and a p3 peptide [57]. β-cleavage of APP mainly occurs in early endosomes [51, 54]. Immunofluorescence experiments in HeLa cells (APP mutant) observed the colocalization of sAPPβ, APP and BACE with early endosomal markers (Rab5) and early endosomal antigen-1 [51]. It has been found that Aβ is accumulated in MVBs and can be released into extracellular space through exosomes [13, 58]. Although only a very small portion of Aβ (<1%) is associated with exosomes, APP, β- and γ- secretase have been detected in exosomes, suggesting that except transport Aβ peptide in the extracellular space, cleavage of APP to generate Aβ could be the main mechanism of spreading lesions [8, 48, 59].

However, the exact role of exosomes in AD progress is still controversial. Several studies have observed that exosomes play a harmful role. A unique Aβ species, tightly binding with GM1, was found in brains of early pathological stage of AD [60]. Endocytic pathway impairment in neurons, including the enlargement of early endosomes and the up-regulation of Rab5 was observed in the brain of a patient with sporadic AD. This impairment significantly accelerated the release of GM1-associated exosomes and induced amyloid fibril formation [61]. Furthermore, exosomes mediate the apoptosis of astrocytes caused by Aβ exposure. Wang and colleagues found that amyloid peptides could activate neutral sphingomyelinase 2 (nSMase2) and induce an increase of PAR-4 and ceramide-containing exosomes secretion in astrocytes. The exosomes were able to be captured by astrocytes and cause apoptosis [62]. Alternatively, fewer amyloid plaques were observed in a mouse model of AD after injection of GW4869, an inhibitor of nSMase2, through prevention of the secretion of exosomes [63]. The protective function of exosomes was also found in various studies. Neuronal exosomes rich in glycosphingolipids could capture Aβ and promote uptake of Aβ by microglia, thus decreasing Aβ and amyloid deposition in APP transgenic mice [64–66]. The cellular prion protein (PrP^C^), a glycosylphosphatidylinositol (GPI)-anchored surface glycoprotein highly expressed on exosomes, was shown to bind oligomeric Aβ42 with high affinity via its flexible N-terminus and accelerate fibrillization of amyloid beta, thereby reducing the neurotoxic effects imparted by oligomeric Aβ [53, 67]. It should be noted that exosomes utilized as protective agents in recent studies almost always come from healthy cells.

In brief, exosomes may serve as a double-edged sword in Aβ metabolism. The imbalanced metabolism of APP may cause accumulation of intracellular Aβ. When beyond the clearance capacity of lysosomes or glial cells, the toxic protein will be released into extracellular space and spread through the brain via the exosome pathway.

## Impact of exosomes on tau pathology

Hyperphosphorylated tau proteins are the major components of NFTs [68]. Tau protein is a member of the family of microtubule-associated proteins encoded by the *MAPT* gene. Because of the alternative splicing of exon 10, there are two major tau isoforms in the adult brain, denoted as 3R and 4R [5, 69]. An abnormal 3R/4R balance is thought to impair the function of tau in keeping stabilization of microtubule structure and material transport [70]. Differences in 3R/4R expression also exist among different diseases. For instance, 3R is the main tau isoform in Pick’s disease, while the 4R tau isoform is a significant component of inclusions in progressive supranuclear palsy (PSP) and corticobasal degeneration (CBD) [71, 72]. In AD, two major tau isoforms are present in the filaments [73]. Tau pathology developed within a definite pattern in AD. The first involved region is entorhinal cortex (Braak stages I-II), then developed to limbic areas (Braak stages III-IV), finally reaches neocortical areas (Braak stages V and IV) [6]. The mechanism of this spreading characteristic of tauopathy throughout human brain has been discussed many years. There is accumulating evidence that tau aggregates spread and replicate in a prion-like manner, with the uptake of pathological tau causing misfolded aggregations of monomeric tau in recipient cells [74, 75]. Exosome-mediated secretion pathways may play an important role in this progress. Studies showed that tau can be exported via an exosome-mediated mechanism in the M1C neuroblastoma tauopathy model, where it is enriched in a phospho-tau biomarker for early AD (AT270). In addition, exosome-associated tau is also present in human CSF samples [76]. Previous studies discovered that propagation of mutant tau between brain regions depended on the presence of microglia, the resident phagocytes of the brain. Microglia spread tau via exosome secretion and depletion of microglia or inhibition of exosome synthesis significantly reduced tau propagation in vitro and in vivo [47]. Polanco and colleagues detected tau in exosomes from tau transgenic rTg4510 mice, and these vesicles were capable of seeding tau aggregation in a threshold-dependent manner [32].

## Conclusions

Increased attention has been paid to the prion-like mechanism involved in the propagation of AD. In this review, we have illustrated the biogenesis and function of exosomes and their impact on amyloidogenic processing and tau pathology. The exosomes pathway may have a “double-edged sword” effect on the process of AD. And the effect is dependent on the cell origins of exosomes and the conditions when exosomes formed. The identification of exosomal pathways could provide not only important insights in the pathogenesis of AD, but due to the tissue-specificity and non-immunogenicity of exosomes, could also serve as an ideal platform for delivery of therapeutic drugs. Furthermore, the molecules packaged in exosomes can be secreted into a variety of bodily fluids, which may serve as biomarkers of disease. Despite the potential benefits of exosomes in diagnosis and therapy, there are some remaining issues, such as making improvements in exosomal isolation techniques and developing a more thorough understanding of the role of exosomes from different cell types under different conditions, which should be the focus of future studies.
